# Motor excitability during visual perception of known and unknown spoken languages

**DOI:** 10.1016/j.bandl.2013.03.002

**Published:** 2013-07

**Authors:** Swathi Swaminathan, Mairéad MacSweeney, Rowan Boyles, Dafydd Waters, Kate E. Watkins, Riikka Möttönen

**Affiliations:** a Department of Experimental Psychology, University of Oxford, South Parks Road, Oxford OX1 3UD, United Kingdom; b Department of Psychology, University of Toronto, Mississauga, Ontario L5L 1C6, Canada; c Institute of Cognitive Neuroscience, University College London, 17 Queen Square, London WC1N 3AR, United Kingdom

**Keywords:** Bilingualism, Lipreading, Motor cortex, Action observation, Motor evoked potentials, Social cognition, Speech, Speechreading, Transcranial magnetic stimulation

## Abstract

It is possible to comprehend speech and discriminate languages by viewing a speaker’s articulatory movements. Transcranial magnetic stimulation studies have shown that viewing speech enhances excitability in the articulatory motor cortex. Here, we investigated the specificity of this enhanced motor excitability in native and non-native speakers of English. Both groups were able to discriminate between speech movements related to a known (i.e., English) and unknown (i.e., Hebrew) language. The motor excitability was higher during observation of a known language than an unknown language or non-speech mouth movements, suggesting that motor resonance is enhanced specifically during observation of mouth movements that convey linguistic information. Surprisingly, however, the excitability was equally high during observation of a static face. Moreover, the motor excitability did not differ between native and non-native speakers. These findings suggest that the articulatory motor cortex processes several kinds of visual cues during speech communication.

## Highlights

• Native and non-native English speakers can visually discriminate English from an unknown language.
• Viewing known speech excites the articulatory motor cortex more than unknown speech.
• Viewing known speech excites the articulatory motor cortex more than non-speech mouth movements.
• Motor excitability is high during observation of a face not speaking.
• Motor excitability does not differ between native and non-native speakers.

## 1 Introduction

Viewing a speaker’s articulatory movements influences speech perception. The well-known McGurk effect demonstrates that seeing an incongruent visual speech signal can modify perception of a clear auditory speech signal. For example, an auditory /ba/ and visual /ga/ are often heard as /da/ (McGurk & MacDonald, 1976). In everyday life, viewing a speaker’s articulatory movements improves speech comprehension under challenging auditory circumstances, such as in noisy environments (Sumby & Pollack, 1954). However, visual speech signals are not as intelligible as auditory speech signals. For example, consonants that share the same place of articulation are hard to discriminate from each other visually (e.g., bilabials /b/, /p/, and /m/) (see, e.g., MacDonald & McGurk, 1978). Speechreading (or “lipreading”) is, therefore, a demanding skill and the ability to understand visual speech varies greatly between people (MacLeod & Summerfield, 1990).

Observation of the speaker’s articulatory movements facilitates learning of non-native phonetic contrasts (Hardison, 2005; Hazan, Sennema, Iba, & Faulkner, 2005; Hirata & Kelly, 2010). Studies have shown that non-native speakers use visual cues during audiovisual speech perception of their L2 and that they can learn to use these cues as efficiently as native speakers (Hardison, 1999, 2003; Hazan et al., 2006; Wang, Behne, & Jiang, 2008). There is also evidence that visual cues can enable non-native speakers to make phonetic distinctions that they are not sensitive to when only auditory cues are available (Navarra & Soto-Faroco, 2007).

It is possible to discriminate languages by viewing a speaker’s articulatory movements. It has been shown that adults can visually discriminate spoken languages as long as at least one of the languages is their first (L1) or second (L2) language (Ronquest, Levi, & Pisoni, 2010; Soto-Faroco et al., 2007). Most likely, visual identification of languages is based on extracting both lexical and rhythmic information from visual speech signals (Ronquest et al., 2010). Interestingly, 4-month old monolingual and bilingual infants are able to discriminate spoken languages visually (Weikum et al., 2007). This skill, however, disappears in monolingual, but not bilingual, infants by the age of 8 months, suggesting that bilingualism enhances visual discrimination abilities in infancy (Sebastián-Gallés, Albareda-Castellot, Weikum, & Werker, 2012; Weikum et al., 2007). Nevertheless, both monolingual and bilingual adults are typically able to extract visual cues from their native or non-native languages and discriminate languages visually (Ronquest et al., 2010; Soto-Faroco et al., 2007).

Numerous neuroimaging studies have shown that viewing a speaker’s articulatory gestures activates the superior temporal cortex and the inferior frontal and premotor regions (Calvert & Campbell, 2003; Hall, Fussell, & Summerfield, 2005; Paulesu et al., 2003; Pekkola et al., 2006; Turner et al., 2009). Importantly, viewing linguistically meaningless mouth movements, i.e., gurns, does not activate these fronto-temporal regions as strongly as viewing speech movements (Campbell et al., 2001; Hall et al., 2005; Turner et al., 2009). The frontal activity has been suggested to reflect involvement of the articulatory motor system in speechreading (e.g., Campbell et al., 2001; Paulesu et al., 2003).

The role of the articulatory motor system in speech perception is under active investigation and its importance is under debate (Hickok, 2010; Hickok, Houde, & Rong, 2011; Pulvermüller & Fadiga, 2010; Scott, McGettigan, & Eisner, 2009). One of the central claims of the motor theory of speech perception is that speech perception and production are tightly linked (Liberman, Cooper, Shankweiler, & Studdert-Kennedy, 1967; Liberman & Mattingly, 1985). According to this theory the speaker’s intended articulatory gestures (or “neuro-motor commands”) are detected from the acoustic speech signal. This inverse (from sensory to articulatory-motor) modeling has been hypothesized to activate the motor brain areas that are important for controlling articulatory movements. Indeed, such activations have been found in some neuroimaging studies during listening to speech (Pulvermüller et al., 2005; Wilson, Saygin, Sereno, & Iacoboni, 2004). Interestingly, listening to non-native speech sounds activates the motor brain regions more strongly than listening to native speech sounds, whereas native speech sounds elicit stronger activity in the superior temporal regions (Callan, Jones, Callan, & Akahane-Yamada, 2004; Wilson & Iacoboni, 2006). This suggests that enhanced articulatory-motor processing complements auditory processing of non-native speech sounds that are more ambiguous than native speech sounds. Given that access to articulatory information is more direct during visual than auditory speech perception, it is plausible that the articulatory motor cortex is engaged in visual speech perception, perhaps even more strongly than in auditory speech perception. No previous studies have investigated differences in articulatory-motor processing of native and non-native visual speech.

Transcranial Magnetic Stimulation (TMS) provides a powerful tool to investigate the excitability of the articulatory motor system during speech perception (for a review, see Möttönen and Watkins 2012). Previous studies using TMS have shown that viewing and listening to speech enhances excitability of the lip representation in the left primary motor cortex (M1) (Fadiga, Craighero, Buccino, & Rizzolatti, 2002; Murakami, Restle, & Ziemann, 2011; Sundara, Namasivayam, & Chen, 2001; Watkins, Strafella, & Paus, 2003). These studies found that the excitability of the articulatory motor system is higher during observation of visual speech than during observation of visual noise (Murakami et al., 2011; Watkins et al., 2003), a fixation dot (Murakami et al., 2011), eye and brow movements (Watkins et al., 2003), or lateral jaw movements (Murakami et al., 2011). These control conditions either involved viewing non-biological stimuli or biological movements that are not performed by using the articulators. Therefore, the specificity of the enhanced excitability in the articulatory motor cortex during visual speech perception is unclear. If this enhanced excitability is speech-specific, viewing speech-related lip movements should excite the articulatory motor cortex more strongly than non-speech lip movements (i.e., gurns). Furthermore, if the articulatory motor cortex is involved in linking perceived articulatory gestures to internal motor codes and extracting linguistic cues from visual speech, excitability should be higher during observation of speech movements related to a known language than to a foreign language, which the observer is not experienced in producing and, which does not convey linguistic information to the observer.

In the present study, we used behavioral tasks and TMS to investigate processing of visual speech in native and non-native speakers of English. Using behavioral tasks, we measured visual language discrimination (English vs. Hebrew) and speechreading (English) skills. We aimed to determine whether our participants are able to discriminate languages visually and how language background (i.e., English as L1 or L2) influences visual speech perception skills. We also used TMS to examine the excitability of the articulatory motor cortex during observation of a known language (i.e., English), an unknown language (i.e., Hebrew), gurns and a still face. As a control, we examined the excitability of the hand motor cortex. The main aim of the TMS experiments was to examine the specificity of the excitability changes in the articulatory motor cortex. We hypothesized that the excitability in the articulatory motor cortex, but not in the hand motor cortex, is higher during observation of a known spoken language than an unknown spoken language or non-speech mouth movements. Furthermore, we aimed to determine whether the modulations of the motor excitability differ between native and non-native English speakers.

## 2 Materials and methods

### 2.1 Participants

Forty six healthy, right-handed adults participated in the study. Data from four additional participants were excluded from analysis due to unreliable MEP or artefacts in the EMG recordings. Handedness was tested using the Edinburgh Handedness Inventory (Oldfield, 1971).

Twenty four participants were native British English speakers (nine men); their mean age was 22 years (18–33 years). Native speakers were defined in this study according to the following criteria: (a) both parents spoke English at home and (b) the primary language in which they received their education up to the age of 18 years was English. Twelve native speakers were assigned to the Lip experiment and 12 were assigned to the Hand experiment.

Twenty two participants were non-native English speakers (nine men); their mean age was 26 years (20–35 years). They spoke 15 different languages as their native language. The non-native group was defined as (a) both parents spoke a language other than English at home, (b) at least until the age of 18 years, the participant had lived outside the United Kingdom in a country where English is not the dominant language, (c) the primary language in which they received education until the age of 18 years was not English. However, non-native English speakers were recruited from the UK and were, therefore, proficient enough in English to engage in employment or full time study in the UK. Average time spent as resident in the UK was 44 months. On average the non-native speakers had started learning English at the age of 11 years and estimated having become fluent at the age of 19 years. Nine non-native speakers were assigned to the Lip experiment and 13 were assigned to the Hand experiment.

All participants reported being unfamiliar with Hebrew and languages related to Hebrew such as Arabic. All participants had normal hearing and normal or corrected to normal eyesight and were screened prior to participation for contraindications to TMS. The study was performed under permission from the National Research Ethics Service.

### 2.2 Electromyography

In all participants electromyography (EMG) activity from the orbicularis oris (OO) muscle was recorded using two surface electrodes (22 ^*^ 30 mm ARBO neonatal electrocardiogram electrodes) attached on the right corners of the lower and upper lip. EMG was also recorded from the first dorsal interosseus (FDI) muscle in the right hand using a pair of electrodes placed on the belly and tendon of the muscle. The raw EMG signal was amplified (gain: 1000), bandpass filtered (10–1000 Hz), and sampled (5000 Hz) using an amplifier, an analog-to-digital converter and a computer running Spike2 software (version 3, Cambridge Electronic Design).

### 2.3 Single pulse transcranial magnetic stimulation

Monophasic TMS pulses were generated by a Magstim 200 stimulator and applied using a 70-mm figure-eight coil (Magstim Co.). The lip area of left M1 was stimulated in the Lip experiment, and hand area of left M1 in the Hand experiment. The position of the coil was adjusted until a robust motor evoked potential (MEP) was observed in the contralateral target muscle. During the experiment the inter-pulse interval was 5–8 s. In this study, we applied single TMS pulses over the motor cortex simply to elicit MEPs in the target muscle. The MEPs provide a direct measure of the excitability of the motor pathways connecting the muscles with their cortical representations. The MEPs are large, when the motor excitability is high.

In the Hand experiment, stimulation intensity was determined as the lowest intensity that elicited robust MEPs (with mean amplitude of approximately 1 mV) in the resting FDI muscle in 10 consecutive trials. In the Lip experiment, stimulation intensity was set as the lowest intensity eliciting robust MEPs (with amplitude of at least 0.2 mV) in 10 consecutive trials in the resting OO muscle. The mean stimulator intensity (±SEM) used to elicit MEPs was 53.7% (±1.57) in the Hand experiment and 65.3% (±1.7) in the Lip experiment (of the maximum stimulator output).

### 2.4 Stimuli in TMS experiments

**Fig. 1:**
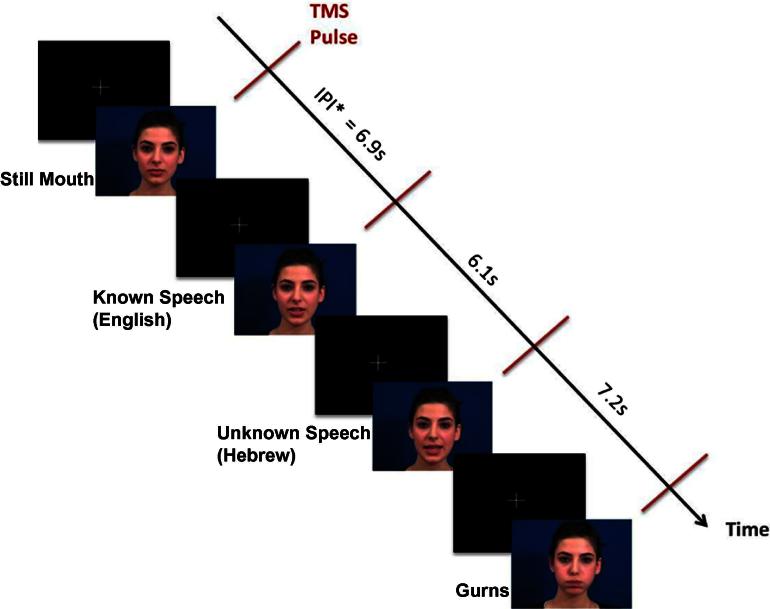
Design of the TMS experiment. Four types of videos were presented in random order: still mouth, known speech, unknown speech and gurns. During each video, one TMS pulse delivered either over the lip or hand representation in the left motor cortex. Inter-pulse-interval (IPI) varied randomly between 5 and 8 s.

All videos were filmed with a female bilingual (English and Hebrew) speaker. In all clips the speaker’s face from the base of her neck to the top of her head were shown (see Fig. 1). Both Lip and Hand experiments included four different stimulus types: (1) *Known Speech* (i.e., English sentences), (2) *Unknown Speech* (i.e., Hebrew sentences), (3) *Gurns* (i.e., sequences of non-speech movements performed by the lips; the sequences ranged from having a single mouth movement repeated over to five different non-speech movements without repetition), (4) *Still mouth* (i.e., video clips of a speaker being silent; these clips included some natural eye blinks). The English sentences were taken from the Bamford–Kowal–Bench standard sentence lists (Bench, Kowal, & Bamford, 1979); these sentences were also translated to Hebrew. During the experiment 40 different stimuli belonging to Known speech, Unknown speech and Gurns conditions were presented. Also, five different Still mouth videos were presented eight times. The clips ranged between 2.5 and 3.5 s in duration with average clip durations matched across the four conditions. Although natural audio-visual speech was recorded, all videos were presented without the audio track.

The videos were presented in eight blocks of 20 clips on a 14-in. Dell monitor using Presentation software (NeuroBehavioral Systems). Each block included five videos from each of the four conditions presented in random order. One TMS pulse was delivered 1.5–2.5 s after the onset of each video. A fixation cross was presented for 3.5 s between the videos.

Participants were instructed to pay attention to the speaker’s mouth and keep their lips and hand relaxed during the TMS experiment. They were also told that the model would be speaking English and another language.

### 2.5 Visual language discrimination task

Eighty videos of the speaker speaking English and Hebrew sentences were presented on the Dell monitor using Presentation software. The videos were the same as used during the TMS experiment. Participants were instructed to click the left mouse button if they thought the language spoken on a video was English and the right mouse button if they thought it was another language. There was an interval of 3.5 s between the videos.

### 2.6 Language Experience and Proficiency Questionnaire (LEAP-Q)

The LEAP-Q (Marian, Blumenfeld, & Kaushanskaya, 2007) is a questionnaire designed to acquire information about linguistic background and is suitable for use with monolinguals and bilinguals. For example, information regarding number of years spent in the UK and age of acquisition of L1 and L2 was obtained.

### 2.7 Reading test

The Vernon-Warden revised reading test (also known as the Kirklees reading test; Hedderly, 1996) was administered to all participants as a measure of English language proficiency. The test contains incomplete sentences that must be completed by selecting the correct word from a list of options. A time limit of 10 min is set.

### 2.8 Speechreading test

The Test of Adult Speechreading (TAS; Mohammed, Campbell, MacSweeney, Barry, & Coleman, 2006; Mohammed et al., 2005) is a standardized English speechreading test. During the test, participants are tested on speechreading accuracy for English words, sentences and stories. The maximum speechreading score is 45.

### 2.9 Procedure

After giving informed consent and filling the screening forms, participants first completed the Vernon Warden reading test and the LEAP-Q. Once these measures were completed, EMG electrodes were attached and the participants completed the TMS experiment. Then, participants completed the visual language discrimination task and the TAS.

### 2.10 Behavioral data analysis

Performance on the visual language discrimination task was analyzed using signal detection principles. The *d*′ measure was calculated for each participant and one-sample *t*-tests was used to test if these values differed significantly from zero (i.e., chance performance). In addition, an independent-samples *t*-test was run to determine whether language discrimination ability differed between native and non-native English speakers.

Independent samples *t*-tests were run to determine differences between the native and non-native English speakers in speechreading ability measured using the TAS, and reading ability measured using the Vernon-Warden test. Finally, correlation analysis was run on the *d*′ scores with the TAS and reading scores.

### 2.11 TMS data analysis

Peak-to-peak amplitude was used as the measure of MEP size. As expected, lip MEPs had shorter onset latency than the hand MEPs because the corticobulbar pathway to the lip muscle is shorter than corticospinal pathway to the hand muscle (Möttönen & Watkins, 2012). Amplitudes of lip MEP were calculated using the time window of 0.01–0.035 s after each TMS pulse. A time window of 0.02–0.04 s after the pulse was used for the hand MEPs.

For each participant, mean MEPs were calculated for each condition. Only MEPs that fell within two standard deviations of the condition mean were included in the analysis and mean MEPs were recalculated. The overall mean and standard deviations of MEPs across conditions was also calculated. MEPs in each condition were standardized to this overall mean, yielding a *z*-score for each condition.

Statistical analyses were carried out using SPSS (version 17.0, IBM). A three-way analysis of variance (ANOVA) was run on the *z*-scores with Stimulus type (Known speech, Unknown speech, Gurns, Still mouth) as a within-subjects factor, and Experiment (Lip, Hand) and Language group (Native, Non-native English speakers) as between subject factors. We also ran separate two-way ANOVAs for Lip and Hand experiments. Post hoc paired *t*-tests comparing different stimulus types were also conducted.

## 3 Results

### 3.1 Behavioral results

**Fig. 2:**
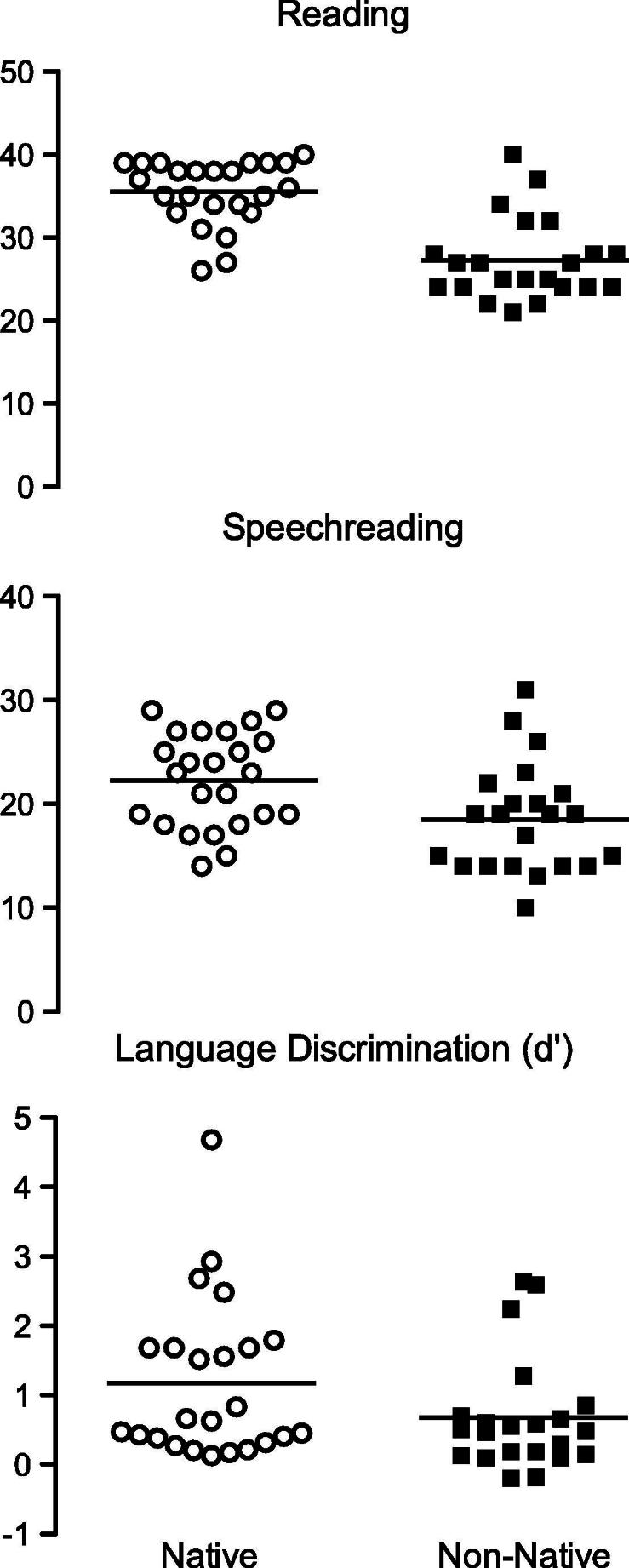
Behavioral results. Performance of native (*n* = 24) and non-native (*n* = 22) English speakers in three behavioral tasks: reading, speechreading and language discrimination.

Reading, speechreading and language discrimination scores of native and non-native speakers of English are presented in Fig. 2. The native English speakers performed better than non-native speakers in the reading assessment (*t*(44) = 6.29, *p* < .001) and in the speechreading test (*t*(44) = 2.63, *p* < .05). There was no significant difference between the performance of native and non-native speakers in the language discrimination task (*t*(44) = 1.70, *p* = .1), although there was a trend towards the native speakers being more accurate than non-native speakers. Both native (*t*(23) = 5.07, *p* < 0.001) and non-native (*t*(21) = 3.90, *p* < 0.05) speakers of English performed better than chance in this task, indicating that the participants were able to discriminate English from Hebrew visual speech. There were no differences in language discrimination, speechreading and reading abilities between participants in the Lip and Hand experiments.

There was a significant positive correlation between visual language discrimination ability and speechreading ability both in the native English group (*r*(22) = 0.53, *p* = 0.008) and in the non-native group (*r*(20) = 0.642, *p* = .001). Reading ability did not correlate with language discrimination or with speechreading ability in either group, or when the groups were combined.

### 3.2 TMS results

**Fig. 3:**
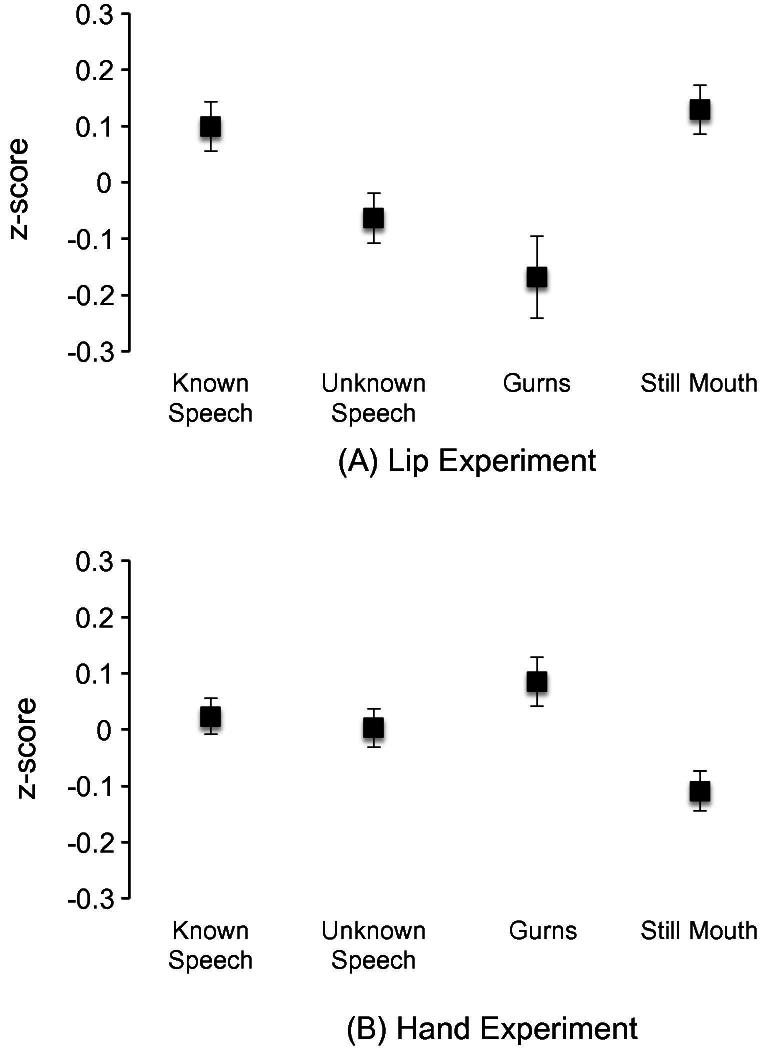
TMS results. Standardized amplitudes of MEPs measured from the lip (A) and hand (B) muscles during observation of known speech (i.e., English), unknown speech (i.e., Hebrew), gurns and still mouth. Differences in MEP amplitudes between conditions reflect differences in the excitability of the lip and hand representations in the motor cortex.

Fig. 3 presents the standardized MEP sizes, i.e., *z*-scores. A three-way ANOVA was conducted: Stimulus type, Experiment and Language group. This revealed a significant Stimulus type ^*^ Experiment (Lip vs. Hand) interaction (*F*(3, 126) = 7.98, *p* < .001 (Greenhouse–Geisser corrected)). No significant main effects or interactions involving Language group (Native vs. Non-native) were found.

A two-way ANOVA for the Lip experiment showed a significant main effect of Stimulus type (*F*(3, 57) = 4.89, *p* < .01). The main effect of Language group and the interaction between Stimulus type and Language group were non-significant. This indicates that the Stimulus type modulated the excitability of the motor lip representation in native and non-native speakers of English in a similar manner. Therefore, we combined these groups in following analyses. Excitability of the motor lip area was higher when the participants observed Known speech (English) than when they observed Unknown speech (Hebrew) (*t*(20) = 4.06, *p* = .001) or Gurns (*t*(20) = 2.34, *p* < .05). The Unknown speech and Gurn did not differ from each other. The motor excitability was higher during observation of Still mouth than during observation of Unknown speech (*t*(20) = 2.51, *p* < .05) or Gurns (*t*(20) = 3.17, *p* < .01). Still mouth and Known speech did not differ from each other.

A two-way ANOVA for the control Hand experiment also showed a significant main effect of Stimulus type (*F*(3, 69) = 3.66, *p* < .05). The main effect of Language group and the interaction between Stimulus type and Language group were non-significant. This indicates that the stimuli modulated the excitability of the motor hand representation in native and non-native speakers of English in a similar manner. Therefore, we combined these groups in following analyses. The excitability of the motor hand representation did not differ between Known speech, Unknown speech and Gurns. However, the excitability was lower during observation of Still mouth than during observation of Gurns (*t*(24) = −2.91, *p* < .01) or Unknown speech (*t*(24) = −2.73, *p* < .05).

## 4 Discussion

The behavioral results indicate that both native and non-native English speakers were able to visually discriminate languages at a level significantly higher than chance. This replicates previous findings (Ronquest et al., 2010; Soto-Faroco et al., 2007) using a new language pair: English and Hebrew. Differences in speech rhythms are likely to be an important cue in visual language discrimination. Ronquest et al. (2010) showed that it is possible to discriminate between stress-timed (i.e., English) and syllable-timed (i.e., Spanish) languages using rhythmic information. English and Hebrew are both stress-timed languages, but the dominant stress patterns are different. English is a trochaic language (i.e., strong–weak pattern is most frequent), whereas Hebrew is an iambic language (i.e., weak–strong pattern is most frequent) (see e.g., Segal, Nir-Sagiv, Kishon-Rabin, & Ravid, 2009). It is possible that the participants used subtle differences in speech rhythms to discriminate these two languages.

Our findings also demonstrate that visual language discrimination ability was positively correlated with English speechreading ability. This suggests that both tasks measure the ability to extract linguistic cues from visual speech. Native speakers performed significantly better than the non-native speakers on the English speechreading test. However, the groups did not differ significantly on the visual language discrimination task, but nonetheless there was a trend in the same direction, i.e., native speakers performed better than non-native speakers. As expected, the native speakers performed better in the reading test that assessed English language proficiency. Interestingly, however, reading ability did not correlate with accuracy in either visual speech perception task. This suggests that some non-native speakers, who had relatively low language proficiency, were relatively good in extracting visual cues from English visual speech. In sum, the behavioral results demonstrated that both native and non-native English speakers were able to extract visual cues from English speech.

The main aim of the TMS experiments was to examine the specificity of the excitability changes in the articulatory motor cortex during observation of a speaker’s face. We found that the lip MEPs were smaller during observation of non-speech mouth movements (i.e., gurns) than articulatory movements related to a known language (i.e., English). This finding supports the hypothesis that the enhanced excitability of the articulatory motor system is speech-specific. The result is in line with the results of Murakami et al. (2011) who found enhanced lip MEPs during speech observation relative to observation of lateral jaw movements. This result is also in line with findings from imaging studies showing increased inferior frontal activations when viewing speech compared to gurns (Hall et al., 2005; Turner et al., 2009). Also, as hypothesized, the lip MEPs were significantly larger during visual perception of a known than an unknown language, providing further evidence for the specificity of the enhanced excitability in the articulatory motor cortex. There are several, not necessarily independent, explanations for this important finding. First, it is possible that viewing a familiar speech rhythm contributed to the enhanced excitability of the articulatory motor cortex during observation of English visual speech. Second, it has been suggested that motor activation during perception is established by association learning (Heyes, 2010). During speech production we can hear (and sometimes see) our own speech, thereby establishing an association between production and perception. Such an association can be established only if the appropriate experience is available. All participants in the present study had experience in producing and perceiving English, but not Hebrew. The difference in the excitability of the articulatory motor cortex may, therefore, reflect differences in the strength of sensorimotor associations for English and Hebrew speech. We propose that the enhanced sensorimotor resonance during visual perception of a known language facilitates linguistic processing. However, further studies, using for example repetitive TMS to disrupt functioning of the articulatory motor cortex, are needed to investigate the causal role of the sensorimotor processes in speechreading.

Importantly, the control experiment demonstrated that the excitability of the hand motor cortex did not differ during observation of known speech, unknown speech and gurns. This indicates that changes in motor excitability are somatotopic during visual speech perception and is in agreement with previous studies (Murakami et al., 2011; Watkins et al., 2003). Observation of communicative hand movements has been shown to enhance excitability in the hand motor cortex in the left hemisphere (Möttönen, Farmer, & Watkins, 2010).

Surprisingly, no significant differences in the excitability of the articulatory motor cortex were found between viewing the still mouth and English speech. The excitability of the articulatory motor cortex was higher when viewing the still mouth than gurns and unknown speech. Furthermore, the excitability of the hand motor cortex was lower when viewing a still mouth than speech and gurns. These results were unexpected in the light of previous imaging studies that found stronger activity in the inferior frontal cortex during observation of speech than a still face (Campbell et al., 2001; Paulesu et al., 2003;). This discrepancy may be partly due to the differences in sensitivity to changes in motor excitability between fMRI and TMS (see Möttönen & Watkins, 2012). There are several possible explanations for enhanced excitability during observation of a still face. First, in the current experiment it was difficult to predict whether the still face would start to speak, because the still face videos were randomized with the speech videos. During the initial 0.5 s of each speech video, the speaker remained still. Possibly, participants were anticipating speech when seeing the still face and, therefore, the excitability of the articulatory motor cortex was enhanced. Second, the still face is a communicative signal. In the monkey brain, there are neurons in the ventral prefrontal cortex that respond more strongly to faces than to non-faces and code gaze, expressions and identity information (e.g., Tsao, Schweers, Moeller, & Freiwald, 2008; Romanski & Diehl, 2011). These neurons have been suggested to be important for social communication (Romanski, 2012). In humans, another person’s still face conveys information that is important for turn taking during face-to-face conversations. Stillness of a conversational partner usually indicates one’s turn to speak. It is possible that this association between seeing a still face and speech production underlies the enhancement of motor excitability. Thus, our results are in line with the idea that the sensorimotor processes are important for controlling conversations (Scott, McGettigan, & Eisner, 2009).

Our TMS results revealed no differences in motor excitability between native and non-native English speakers during visual perception of spoken languages. The results suggest that the articulatory motor system is engaged in a similar manner during visual perception of spoken language in native and non-native speakers. The lack of differences in motor excitability may seem surprising in the light of behavioral results, showing a native-speakers advantage in the speechreading task and a similar (non-significant) trend in the language discrimination task. There are several possible explanations for the lack of difference in motor excitability between the groups. First, it should be noted that the groups did not differ significantly in their ability to discriminate English and Hebrew sentences and that this task was difficult for both groups. Although the performance was better than chance in both native and non-native groups, it was still rather poor in both groups (mean *d*′ values of around 1). Thus, the stimuli used in the TMS experiment were equally difficult for native and non-native speakers. Second, in the current study the motor excitability was measured during passive observation of the stimuli, not during active visual speech perception tasks. Further studies are needed to examine whether motor excitability is higher during active speechreading than passive viewing of a speaker’s articulatory movements and whether motor excitability would differ between native and non-native speakers during active speechreading. Third, a large fronto-temporal network of brain areas is involved in speechreading (Calvert & Campbell, 2003; Hall et al., 2005; Paulesu et al., 2003; Pekkola et al., 2006; Turner et al., 2009). The articulatory motor cortex is only one node in this network and it is possible that other areas are more sensitive to differences in speechreading ability. For example, activity in the left superior temporal gyrus and posterior cingulate cortex during visual perception of spoken sentences has been shown to positively correlate with speechreading scores (Hall et al., 2005). Fourth, a larger sample size may be needed to reveal subtle differences in motor excitability between native and non-native speakers. In sum, although the present study did not reveal any differences in motor excitability between native and non-native speakers during visual perception of spoken languages, further studies are needed to investigate the effect of language background on involvement of the articulatory motor system in speech perception. For example, studies investigating perception of auditory, visual and audiovisual speech signals in a large number of participants that differ in their age of acquisition and proficiency of the second language are needed.

In summary, our findings show that visual perception of a face speaking a known language or a face not speaking enhances excitability of the articulatory motor cortex relative to visual perception of a face that speaks an unknown language or performs non-speech movements with his/her mouth. Thus, visual signals that the perceiver is experienced in producing and perceiving during face-to-face conversations enhance excitability in the articulatory motor cortex. The enhanced sensorimotor resonance during visual perception of known speech (relative to unknown speech and gurns) is likely to support extraction of linguistic (e.g., phonetic and rhythmic) cues. On the other hand, the relatively high motor excitability during observation of a face not speaking suggests that the articulatory motor cortex also processes non-linguistic visual cues during speech communication. The speaker’s silent face is an important communicative cue that can be used, for example, to control turn taking in conversations. Altogether, the findings suggest that sensorimotor processes may have multiple functions in visual perception of a speaker’s face during face-to-face communication. Future studies should aim to further characterize how the articulatory motor cortex contributes to extraction of both linguistic and non-linguistic cues from the speaker’s face.
